# Revisiting the Antigen 85 Complex as a Target for Functional Antibody-Based Strategies Against *Mycobacterium tuberculosis*

**DOI:** 10.3390/microorganisms14040863

**Published:** 2026-04-11

**Authors:** Izat Smekenov, Nazym Tleumbetova, Sagit Bektassov, Amangeldy Bissenbaev

**Affiliations:** 1 Department of Molecular Biology and Genetics, Faculty of Biology and Biotechnology, Al-Farabi Kazakh National University, Almaty 050040, Kazakhstan; smekenovizat@gmail.com (I.S.); nn_nazym@mail.ru (N.T.); 2 Scientific Research Institute of Biology and Biotechnology Problems, Al-Farabi Kazakh National University, Almaty 050040, Kazakhstan; 3 Reference Laboratory for the Control of Especially Dangerous Infections, Almaty 050008, Kazakhstan; 4 National Scientific Center of Phthisiopulmonology of the Ministry of health of the Republic of Kazakhstan, Almaty 050010, Kazakhstan; sagit_bektasov@mail.ru

**Keywords:** *Mycobacterium tuberculosis*, antigen 85 complex, Ag85B, nanobodies, mycolyltransferase, cell wall biosynthesis, antibody-mediated sensitization, combination therapy

## Abstract

The antigen 85 (Ag85) complex of *Mycobacterium tuberculosis* comprises secreted mycolyltransferases essential for cell wall biosynthesis and envelope integrity. Although extensively studied as immunodominant antigens and targets of small-molecule inhibitors, their potential for antibody-mediated functional modulation remains underexplored. In this review, we re-evaluate the Ag85 complex from a mechanistic and therapeutic perspective, focusing on its accessibility, structural organization, and functional vulnerabilities within the mycobacterial cell envelope. We highlight the distinction between antigen recognition and functional modulation, noting that conventional antibody responses often target immunodominant but functionally irrelevant epitopes. We propose that nanobodies represent a promising platform for targeting conformationally or spatially restricted regions of Ag85 enzymes that are not readily accessible to conventional antibodies. Based on structural and biochemical insights, we outline a framework in which Ag85-targeting nanobodies act as sensitizing agents that perturb cell wall biosynthesis and enhance susceptibility to antibiotics or cell wall-degrading enzymes. We further discuss key challenges, including antigen accessibility under physiological conditions, intracellular localization, and nanobody stability, which may influence the feasibility of this approach.

## 1. Introduction

Tuberculosis (TB) remains one of the leading causes of mortality from infectious diseases worldwide, a situation further aggravated by the continuous emergence and global spread of multidrug-resistant (MDR) and extensively drug-resistant (XDR) *Mycobacterium tuberculosis* strains [1,2]. Despite the availability of combination chemotherapy, TB treatment remains prolonged and complex, with significant challenges related to drug tolerance, treatment adherence, and relapse. These limitations have stimulated a growing interest in non-traditional and adjunctive therapeutic strategies that complement classical antibiotics rather than replace them. Current therapeutic strategies for tuberculosis rely on prolonged multidrug regimens targeting essential bacterial processes such as cell wall biosynthesis, transcription, and energy metabolism. However, the emergence of drug-resistant strains has stimulated the development of alternative approaches, including host-directed therapies, immunomodulatory strategies, and combination regimens designed to enhance bacterial susceptibility to existing antibiotics. These evolving strategies highlight the need for complementary modalities that can sensitize *Mycobacterium tuberculosis* to antimicrobial stress [3].

A defining feature of *M. tuberculosis* is its exceptionally complex, lipid-rich cell envelope, which acts as a major permeability barrier and contributes substantially to intrinsic antibiotic tolerance and persistence [4,5,6]. Central to the biosynthesis and remodeling of this envelope is the antigen 85 (Ag85) complex, composed of three closely related mycolyltransferases: Ag85A (FbpA), Ag85B (FbpB), and Ag85C (FbpC). These enzymes catalyze key reactions in the transfer of mycolic acids to trehalose and arabinogalactan, leading to the formation of trehalose dimycolate and the mycolyl–arabinogalactan–peptidoglycan complex, both of which are essential for cell wall integrity, virulence, and survival [7,8,9].

Genetic and pharmacological studies have firmly established Ag85 enzymes as validated drug targets. Inhibition of Ag85 activity by small-molecule compounds or substrate analogs disrupts cell wall assembly, alters mycolate composition, and compromises mycobacterial growth and viability [10,11,12]. These findings underscore the functional vulnerability of the Ag85 complex and highlight its central role in maintaining the structural and physiological robustness of the mycobacterial cell envelope.

Beyond their enzymatic importance, Ag85 proteins occupy a unique position at the interface of bacterial physiology and host immunity. They are among the most abundantly secreted and immunodominant antigens of *M. tuberculosis* and have been extensively investigated as vaccine candidates and diagnostic markers [13,14]. In particular, Ag85B elicits strong T-cell responses and is readily detected in culture filtrates and infected tissues, reflecting its extracellular availability and accessibility to host immune components [15,16]. This dual role—as an essential cell wall enzyme and a prominent immune antigen—renders Ag85 an unusually attractive and multifaceted therapeutic target.

While antibodies against Ag85 proteins have been widely generated and characterized for immunological, diagnostic, and vaccine-related purposes, their potential to exert direct functional effects on Ag85 enzymatic activity or mycobacterial physiology has received comparatively limited systematic attention. As discussed in Section 3, this gap reflects a broader principle in antibody–enzyme interactions: immunodominance does not guarantee functional relevance. Most antibodies target surface-exposed loops that tolerate binding without perturbing catalysis, whereas catalytically critical regions may be less immunogenic or sterically shielded.

In this context, single-domain antibodies (nanobodies) offer a distinct alternative. Nanobodies possess unique structural and biophysical properties, including small size, high stability, and the ability to access recessed or conformationally sensitive epitopes, which may enable functional modulation of Ag85 enzymes even in the absence of complete catalytic inhibition. Importantly, partial interference with cell wall biosynthesis or transient destabilization of envelope integrity may be sufficient to sensitize mycobacteria to antibiotics, environmental stress, or cell wall-targeting agents such as bacteriophage-derived lysins [17,18].

**Figure 1:**
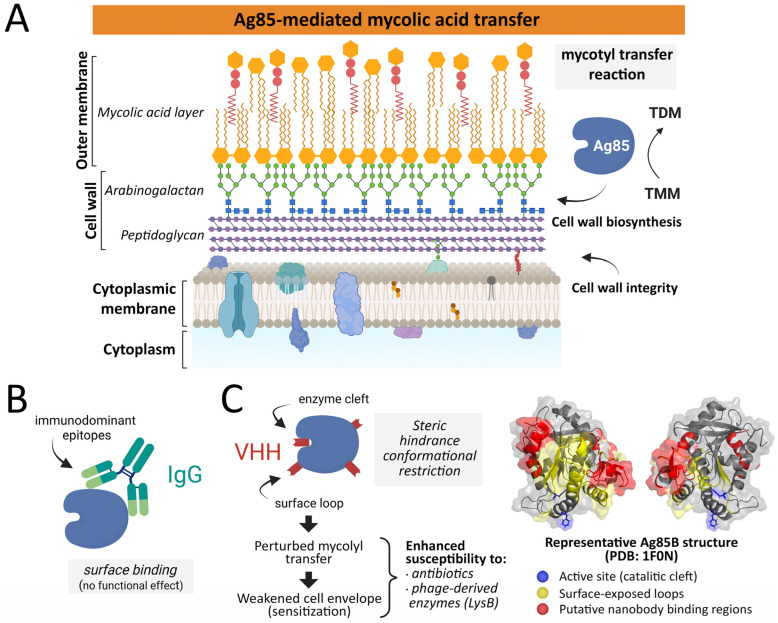
Conceptual framework for nanobody-mediated sensitization of *Mycobacterium tuberculosis* through targeting of the Ag85 complex. (**A**) Ag85-mediated mycolic acid transfer; (**B**) conventional antibody recognition; (**C**) nanobody-mediated functional modulation. The antigen 85 (Ag85) complex mediates the transfer of mycolic acids from trehalose monomycolate (TMM) to trehalose dimycolate (TDM) and arabinogalactan, playing a central role in mycobacterial cell wall biosynthesis. Conventional antibodies typically recognize immunodominant surface-exposed epitopes without significantly affecting enzymatic function. In contrast, nanobodies (variable domain of heavy-chain-only antibodies, VHH) are proposed to access recessed or conformationally sensitive regions of Ag85 enzymes, such as enzyme clefts or surface loops. The binding of nanobodies may induce steric hindrance or conformational restriction that perturbs mycolyl transfer reactions. Partial disruption of Ag85 activity can weaken the integrity of the mycobacterial cell envelope and increase susceptibility to conventional antibiotics or phage-derived cell wall-degrading enzymes such as LysB.

From this perspective, antibody- and nanobody-based approaches targeting Ag85 may be better conceptualized not as standalone antimicrobials, but as adjuvant strategies that weaken the mycobacterial cell envelope and enhance the efficacy of existing treatments. In this review, we synthesize current knowledge on the Ag85 complex at the intersection of mycobacterial cell wall biology, immunogenicity, and therapeutic targeting. We critically examine the evidence for antibody interactions with Ag85 proteins, discuss structural and functional considerations that may enable antibody-mediated modulation of Ag85 activity, and highlight emerging opportunities for nanobody-based and combinatorial strategies against *M. tuberculosis*. The conceptual framework discussed in this review is summarized in Figure 1.

## 2. The Ag85 Complex: Structure, Localization, and Functional Accessibility

The antigen 85 (Ag85) complex of *Mycobacterium tuberculosis* consists of three closely related mycolyltransferases—Ag85A (FbpA), Ag85B (FbpB), and Ag85C (FbpC)—which share high sequence and structural similarity but differ in expression levels, regulation, and relative contribution to cell wall assembly [8,14]. All three enzymes catalyze transesterification reactions involving trehalose monomycolate (TMM), generating trehalose dimycolate (TDM, cord factor) and transferring mycolic acids to the arabinogalactan–peptidoglycan core. These reactions are essential for maintaining the integrity, impermeability, and mechanical stability of the mycobacterial cell envelope [7,9]. The characteristics of the Ag85 complex and their implications are presented in Table 1.

### 2.1. Structural Organization of Ag85 Enzymes

Crystal structures of Ag85 proteins have revealed a conserved α/β hydrolase fold with a well-defined catalytic triad and an extended substrate-binding groove adapted to accommodate long-chain mycolic acids [11,19]. While the active-site architecture is highly conserved across Ag85A, Ag85B, and Ag85C, subtle differences in surface loops and peripheral regions contribute to isoform-specific properties, including substrate preference and interaction with the cell envelope [12]. Importantly, these surface-exposed regions often coincide with immunogenic epitopes, raising the possibility that antibody binding could influence enzyme activity indirectly through steric or allosteric effects.

Notably, structural studies have demonstrated that small-molecule inhibitors of Ag85 frequently bind outside the catalytic center, stabilizing non-productive conformations or interfering with substrate positioning [11,12]. In addition to structural characterization, several small-molecule inhibitors of the Ag85 complex have been identified, acting through diverse mechanisms including covalent inhibition, substrate mimicry, and allosteric modulation. Notably, a number of these compounds bind outside the catalytic center or stabilize non-productive enzyme conformations, thereby interfering with substrate positioning and enzymatic dynamics. Importantly, several inhibitors act through non-catalytic or allosteric mechanisms. Representative examples of Ag85 inhibitors and their mechanisms of interaction are summarized in Table 2. These observations provide a conceptual precedent for functional modulation of Ag85 that does not require direct active-site occlusion, supporting the feasibility of antibody- and nanobody-based strategies.

### 2.2. Localization and Secretion of Ag85 Proteins

Unlike many cytosolic metabolic enzymes, Ag85 proteins are synthesized with N-terminal signal peptides and are actively secreted via the Sec pathway [7,21]. Following secretion, a substantial fraction of Ag85 associates with the periplasmic space and the outer layers of the mycobacterial cell envelope, while another fraction is released into the extracellular milieu. Proteomic analyses of culture filtrates have consistently identified Ag85A/B/C among the most abundant secreted proteins of *M. tuberculosis* [22,23].

This dual localization—both cell-associated and extracellular—distinguishes Ag85 from many other essential bacterial enzymes and has important functional implications. Cell wall-associated Ag85 participates directly in envelope biogenesis, whereas extracellular Ag85 may remain enzymatically active or interact transiently with cell surface substrates. From an immunological perspective, this localization renders Ag85 readily accessible to antibodies, as reflected by its strong immunodominance during infection and vaccination [14].

### 2.3. Functional Accessibility and Implications for Antibody Targeting

The extracellular and surface-associated presence of Ag85 has fueled long-standing interest in antibody-based detection and vaccination strategies. However, accessibility alone does not guarantee functional vulnerability. The extent to which Ag85 enzymes are exposed in a conformation permissive for antibody binding—and whether such binding can perturb catalytic activity or enzyme localization—likely depends on growth conditions, cell wall dynamics, and the local organization of mycolate-containing substrates [5].

Importantly, Ag85 enzymes operate at the interface of hydrophobic lipid substrates and the aqueous extracellular environment, a context that may be particularly sensitive to steric interference. Antibody or nanobody binding to surface-exposed regions involved in substrate docking, membrane association, or protein–protein interactions could plausibly disrupt mycolyl transfer reactions or alter the spatial coordination of cell wall assembly. Even partial or transient interference with these processes may have downstream consequences for envelope integrity, permeability, and susceptibility to stress or antimicrobial agents.

A critical consideration for antibody-based strategies targeting Ag85 is the extent to which these enzymes are accessible under physiologically relevant infection conditions. Although Ag85 proteins are secreted and have been detected both in culture filtrates and in association with the mycobacterial cell envelope [7,21,22,23], their spatial distribution in vivo is likely heterogeneous and dynamic. A significant fraction of Ag85 is thought to localize within the periplasmic space or remain transiently associated with the outer layers of the cell wall, where access by externally applied binding molecules may be partially restricted. Furthermore, the complex architecture of the mycobacterial cell envelope, characterized by a dense and hydrophobic mycolic acid layer, may limit the diffusion and effective engagement of antibodies with enzymatic targets embedded within or beneath this barrier [5]. Even when Ag85 is exposed, its orientation, local concentration, and interaction with lipid substrates may constrain productive binding. These challenges are compounded by the intracellular lifestyle of *Mycobacterium tuberculosis*. Within macrophages, bacteria reside in modified phagosomal compartments, where antibody access depends not only on antigen exposure but also on the ability of the antibody or nanobody to reach the relevant cellular niche [24,25]. While nanobodies may exhibit improved tissue penetration and cellular uptake compared to conventional antibodies, their effective concentration at the site of Ag85 activity remains uncertain.

Taken together, these considerations indicate that Ag85 accessibility is highly context-dependent, varying with the bacterial physiological state, cell wall dynamics, and host environment. This reinforces the need for experimental validation of nanobody-target engagement under conditions that closely mimic the in vivo infection context.

## 3. Antibody Responses to Ag85: Immunodominance, Epitope Distribution, and Functional Limitations

### 3.1. Immunodominance, Epitope Distribution, and Cross-Reactivity

The antigen 85 (Ag85) complex has long been recognized as one of the most immunodominant protein families of *Mycobacterium tuberculosis*. Both humoral and cellular immune responses against Ag85A, Ag85B, and Ag85C are readily detected during natural infection, vaccination, and experimental immunization, making these proteins central components of tuberculosis vaccine research and immunodiagnostic assays [13,14]. Among the three isoforms, Ag85B is often reported as the most abundantly expressed and immunogenic, eliciting strong antigen-specific T-cell and antibody responses [15,16].

Multiple studies have demonstrated that immunization with recombinant Ag85 proteins induces robust antibody responses across different host species, including mice, rabbits, and humans [26,27]. Antibodies recognizing Ag85 are commonly detected in sera from tuberculosis patients and vaccinated individuals, reflecting both the abundance and extracellular availability of these antigens during infection [28,29]. Epitope mapping studies have revealed that antibody responses to Ag85 are typically polyclonal and broadly distributed across the protein surface. Immunodominant regions frequently correspond to surface-exposed loops and flexible regions that are readily accessible to the immune system but are not necessarily involved in catalysis or substrate binding [30,31]. As a result, a large fraction of Ag85-specific antibodies binds efficiently to the antigen without measurably affecting its enzymatic activity or biological function.

The high degree of sequence and structural conservation among Ag85A, Ag85B, and Ag85C further shapes the antibody response. Many antibodies raised against one isoform display substantial cross-reactivity with the other members of the Ag85 family, as well as with homologous proteins expressed by non-tuberculous mycobacteria such as *Mycobacterium smegmatis* [14,26]. While such cross-reactivity is advantageous for diagnostic applications and may contribute to broad immune recognition, it complicates efforts to dissect isoform-specific functional roles or to achieve targeted inhibition of a single Ag85 enzyme.

From a functional standpoint, cross-reactive antibodies are more likely to recognize conserved, structurally stable regions rather than isoform-specific or catalytically critical motifs.

### 3.2. Functional Consequences of Antibody Binding to Ag85

Despite the extensive literature on Ag85 immunogenicity, relatively few studies have addressed whether antibodies against Ag85 exert direct functional effects on mycobacterial physiology. Most anti-Ag85 antibodies have been developed and validated as diagnostic or research reagents, where binding affinity and specificity are prioritized over functional modulation [31,32]. In contrast, systematic assessments of enzyme inhibition, altered substrate processing, or growth phenotypes in the presence of Ag85-specific antibodies remain scarce. This apparent disconnect highlights a general principle in antibody–enzyme interactions: immunodominance does not equate to functional relevance. Antibodies preferentially target regions that are structurally flexible, solvent-exposed, and tolerant to variation, whereas catalytic or regulatory regions may be less immunogenic or sterically shielded. As a result, the majority of antibodies generated against Ag85 bind the protein without perturbing its catalytic cycle or its role in cell wall biosynthesis.

The predominance of non-functional Ag85-directed antibodies does not preclude the existence of rare antibodies capable of modulating enzyme activity. Rather, it underscores the need for selection strategies explicitly designed to enrich for functional binders, rather than for antibodies that merely recognize abundant antigenic surfaces. This distinction is particularly relevant for therapeutic concepts that aim to exploit antibody-mediated interference with essential bacterial processes.

In this context, conventional monoclonal antibodies may be disadvantaged by their size and limited access to recessed or conformationally sensitive regions of Ag85 enzymes. Smaller binding scaffolds, such as single-domain antibodies (nanobodies), offer potential advantages by virtue of their compact size, rigid paratope architecture, and ability to engage epitopes that are inaccessible to classical antibodies [33]. These properties raise the possibility that nanobodies could preferentially target functionally relevant regions of Ag85 or stabilize non-productive enzyme conformations, even in the absence of complete active-site blockade. Notably, a single-domain antibody specific for an HLA-presented epitope derived from the Ag85B antigen has been reported [34], demonstrating the feasibility of generating nanobodies against Ag85-derived sequences in an immunological context. However, this nanobody recognizes a peptide–MHC complex rather than the native Ag85 enzyme and does not address enzyme accessibility or functional modulation at the bacterial surface.

Together, the extensive immunogenicity of Ag85 and the relative paucity of documented functional antibody effects define both a challenge and an opportunity. Understanding why most anti-Ag85 antibodies are non-inhibitory provides a conceptual framework for designing next-generation antibody-based strategies that move beyond antigen recognition toward functional modulation of mycobacterial cell wall biosynthesis. These observations highlight that functional antibody-mediated modulation of Ag85 is likely to be rare and context-dependent, requiring carefully designed selection strategies and experimental validation.

## 4. Nanobodies as Tools to Access Functional Epitopes of Ag85

Conventional monoclonal antibodies have proven highly effective for antigen recognition and immune engagement; however, their large size and flexible architecture can limit access to recessed, transient, or conformationally sensitive epitopes on bacterial enzymes. This limitation is particularly relevant for targets such as the Ag85 complex, whose catalytic function depends on extended hydrophobic grooves, surface-associated docking regions, and dynamic conformational rearrangements during mycolyl transfer reactions [12,19]. In this context, single-domain antibodies (nanobodies, VHHs) offer a distinct and potentially advantageous alternative [35,36,37].

### 4.1. Structural and Biophysical Advantages of Nanobodies

Nanobodies are derived from the variable domains of heavy-chain-only antibodies naturally produced by camelids. Their small size (~15 kDa), rigid β-sheet framework, and elongated complementarity-determining regions enable high-affinity binding to epitopes that are sterically inaccessible to conventional IgG molecules [33]. Importantly, nanobodies frequently recognize concave or buried surfaces, including enzyme clefts and protein–protein interaction interfaces, and can stabilize specific conformational states of their targets [38,39].

These properties have been successfully exploited to generate nanobodies that modulate the activity of diverse enzymes, receptors, and signaling proteins, either by direct steric occlusion or by allosteric mechanisms that restrict conformational flexibility [40,41]. In several cases, nanobodies have been shown to function as conformational traps, locking enzymes into inactive or suboptimal states without directly occupying the catalytic center.

### 4.2. Implications for Targeting Ag85 Enzymes

The structural organization of Ag85 enzymes suggests multiple potential points of vulnerability to nanobody binding. In addition to the catalytic triad, Ag85 proteins contain surface-exposed regions implicated in substrate accommodation, membrane association, and positioning of trehalose-containing acceptors [11,19]. These regions are often distal from the active site yet essential for productive catalysis, making them attractive targets for allosteric interference.

Nanobodies targeting such regions could, in principle, interfere with Ag85 function through several non-mutually exclusive mechanisms: (i) steric hindrance of substrate entry or product release; (ii) disruption of transient interactions between Ag85 and the mycobacterial cell envelope; or (iii) stabilization of non-productive enzyme conformations. Notably, even partial or transient modulation of Ag85 activity may be sufficient to perturb the finely balanced process of cell wall assembly, leading to cumulative defects in envelope integrity. Nevertheless, the extent to which these mechanisms can be achieved under physiological conditions remains uncertain and requires direct experimental validation.

### 4.3. Accessibility and Extracellular Targeting

Unlike many essential bacterial enzymes, Ag85 proteins are secreted and remain associated with the cell wall or extracellular milieu, rendering them accessible to externally applied binding molecules. This characteristic is particularly relevant for nanobody-based strategies that do not rely on intracellular delivery.

Nevertheless, accessibility alone does not guarantee functional impact. Effective nanobody-mediated modulation of Ag85 likely requires precise epitope targeting, emphasizing the importance of selection and screening strategies that prioritize functional outcomes over simple antigen binding. In this regard, display-based technologies, including phage and yeast surface display, provide powerful tools to enrich for nanobodies that recognize Ag85 in conformations and contexts that more closely resemble those encountered at the mycobacterial cell surface.

## 5. Toward Functional Selection Strategies

The relative scarcity of reports describing direct functional effects of antibodies on Ag85 underscores the need for selection paradigms that go beyond affinity and specificity. Functional screening approaches—such as competitive binding assays, conformational probes, or phenotypic readouts related to cell wall integrity—may be required to identify rare nanobodies capable of modulating Ag85 activity. Importantly, such strategies align with the broader shift in antibody discovery from purely binding-based metrics toward functional performance. The conceptual framework discussed in this review is summarized in Figure 1.

In this framework, nanobodies should be viewed not merely as smaller antibodies, but as precision tools capable of interrogating and perturbing specific structural and functional features of essential bacterial enzymes. When applied to the Ag85 complex, nanobody-based approaches offer a rational path to explore antibody-mediated modulation of mycolyltransferase function and to bridge the gap between immunogenicity and functional vulnerability.

The intrinsic resistance of *Mycobacterium tuberculosis* to many antimicrobial agents is largely attributable to its complex and highly impermeable cell envelope. This barrier not only limits antibiotic penetration but also contributes to phenotypic tolerance and persistence, complicating treatment even in the absence of classical resistance mutations [5,12]. Consequently, increasing attention has been directed toward adjuvant strategies that weaken cell envelope integrity and thereby sensitize mycobacteria to existing antimicrobial interventions.

### Combinatorial Strategies: Nanobodies as Adjuvants

Several classes of anti-tubercular antibiotics, including rifamycins, fluoroquinolones, and aminoglycosides, exhibit limited penetration through the mycobacterial cell wall. Alterations in envelope composition or permeability can markedly influence their efficacy [4,42]. Accordingly, interventions that subtly disrupt cell wall organization—without necessarily killing the bacterium outright—can enhance the efficacy of conventional antibiotics. In this context, Ag85-targeting nanobodies may function as cell wall-active adjuvants, increasing envelope permeability or destabilizing lipid-rich layers. Even modest perturbations of cell wall integrity may therefore translate into biologically meaningful improvements in antibiotic access during prolonged tuberculosis treatment.

Bacteriophage-derived enzymes, including endolysins and specialized mycobacteriophage-associated proteins, have emerged as promising non-traditional antimicrobials. Among these, mycobacteriophage D29 Lysin B (LysB) targets ester linkages between mycolic acids and arabinogalactan, directly attacking the integrity of the mycobacterial outer membrane [18,43]. While LysB alone may exhibit limited activity depending on accessibility and experimental conditions, its capacity to weaken the mycolate layer makes it an attractive candidate for combination strategies. Nanobody-mediated perturbation of Ag85 function could enhance the susceptibility of mycobacteria to LysB by altering the dynamics of mycolate attachment and turnover. Conversely, partial degradation of the mycolate layer by LysB may increase the accessibility of Ag85 enzymes or their substrates, creating a positive feedback loop that amplifies the overall effect.

The combinatorial use of nanobodies with antibiotics or phage-derived enzymes offers several conceptual advantages. First, it reduces selective pressure for resistance by avoiding reliance on a single lethal mechanism. Second, it leverages complementary modes of action—structural destabilization, enzymatic interference, and intracellular inhibition—to overcome the multifactorial defenses of mycobacteria. Finally, it aligns with emerging paradigms in antimicrobial development that prioritize sensitization and potentiation over direct bactericidal activity. Importantly, such strategies are particularly well suited to nanobodies, whose stability, modularity, and ease of engineering facilitate their integration into multifunctional or combination-based therapeutic designs [20,44,45]. Despite this conceptual appeal, the magnitude and reproducibility of such synergistic effects remain to be established in biologically relevant models.

## 6. Experimental and Conceptual Challenges

Despite the conceptual appeal of targeting the Ag85 complex with nanobodies, several experimental and conceptual challenges must be considered when evaluating the feasibility and translational potential of this approach.

### 6.1. Target Accessibility and Spatial Context

Although Ag85 proteins are secreted and partially associated with the mycobacterial cell envelope, their functional accessibility to externally applied nanobodies remains context-dependent. The degree of surface exposure of catalytically relevant regions likely varies with growth phase, environmental conditions, and the dynamic remodeling of the cell wall [5]. Moreover, Ag85 enzymes operate at the interface of hydrophobic lipid substrates and the cell envelope, a spatial context that may restrict effective antibody engagement even when antigen binding is detectable.

These considerations highlight the limitation of relying solely on recombinant antigens or in vitro binding assays to infer functional relevance. Binding to purified Ag85 does not necessarily translate into productive engagement of the enzyme in its native, cell-associated state.

### 6.2. Intracellular Localization of Mycobacterium tuberculosis and Macrophage Uptake Considerations

The relevance of target accessibility extends beyond the bacterial cell surface, as *Mycobacterium tuberculosis* is a facultative intracellular pathogen that predominantly resides within macrophages. Following phagocytosis, *M. tuberculosis* persists within modified phagosomal compartments, where it actively subverts normal phagosome maturation and avoids lysosomal degradation [24,46]. This intracellular lifestyle imposes an additional layer of complexity for antibody-based strategies, as conventional antibodies are generally considered ineffective against intracellular pathogens. In this context, nanobodies represent a distinct antibody format for which intracellular considerations become biologically relevant. Due to their small size and favorable biophysical properties, nanobodies can be internalized by macrophages more readily than conventional IgG molecules [25,33]. While spontaneous cytosolic delivery of nanobodies is unlikely, their localization within endosomal or phagosomal compartments may be sufficient to enable interactions with intracellular mycobacteria or with host–pathogen interfaces in the macrophage niche. Importantly, this potential intracellular access should not be interpreted as evidence of direct cytosolic targeting, but rather as a biologically grounded rationale for considering nanobody-based strategies in tuberculosis. The overlap between macrophage uptake pathways and the intracellular residence of *M. tuberculosis* highlights both an opportunity and a challenge, emphasizing the need for realistic expectations and careful experimental validation when evaluating functional outcomes.

### 6.3. Distinguishing Binding from Functional Modulation

A central challenge in antibody discovery against enzymatic targets is the disconnect between antigen recognition and functional impact. As discussed earlier, immunodominant epitopes on Ag85 are frequently located in surface-exposed regions that tolerate antibody binding without perturbing catalysis [30,47]. Consequently, selection strategies based purely on affinity or specificity risk enriching for nanobodies that bind Ag85 efficiently yet lack biological activity.

Functional screening paradigms—designed to detect changes in enzyme activity, cell wall integrity, or bacterial susceptibility—are therefore essential but often technically demanding. In the absence of direct enzymatic assays compatible with the native lipid substrates of Ag85, phenotypic readouts may serve as indirect but biologically relevant proxies.

### 6.4. Model Systems and Translational Relevance

The use of non-pathogenic mycobacteria such as *Mycobacterium smegmatis* offers practical advantages for early-stage screening and hypothesis testing. However, differences in cell wall composition, growth rate, and Ag85 regulation limit the direct extrapolation of findings to *Mycobacterium tuberculosis* [4]. Observations made in surrogate models must therefore be interpreted cautiously and validated under conditions that more closely approximate the pathogenic context. Beyond the choice of bacterial model, additional translational constraints arise from considerations of nanobody delivery and localization in vivo. As *M. tuberculosis* is a facultative intracellular pathogen that resides predominantly within macrophages, the route of administration and the ability of nanobodies to reach relevant cellular compartments become critical determinants of biological relevance. In this regard, pulmonary delivery has been proposed as a potentially advantageous strategy for tuberculosis-focused biologics, as it may enhance local concentrations in the lung while limiting systemic exposure [48]. Nevertheless, factors such as airway deposition, mucociliary clearance, proteolytic stability, and intracellular accessibility within macrophages represent significant barriers that must be addressed experimentally [48,49]. Additionally, nanobody stability, persistence, and effective concentration at the bacterial surface represent important considerations for in vitro and eventual in vivo applications. Together, these factors underscore the need for realistic expectations regarding the magnitude and kinetics of nanobody-mediated effects.

### 6.5. Resistance and Evolutionary Considerations

While nanobody-based strategies are often viewed as less prone to resistance development than classical antibiotics, selective pressure remains an inherent concern. Mutations that alter Ag85 surface features, modulate secretion, or compensate for partial loss of enzymatic efficiency could potentially attenuate nanobody efficacy. However, the essentiality and functional constraints of Ag85 enzymes may limit the evolutionary space available for resistance, particularly when nanobodies are deployed as part of combination regimens rather than as monotherapies.

### 6.6. Stability and Persistence of Nanobodies in Biological Environments

In addition to target accessibility and functional activity, the stability of nanobodies at the molecular and nanoscale levels represents an important consideration for their therapeutic application [35,39,50]. Nanobodies are generally characterized by high intrinsic stability, including resistance to thermal denaturation and the ability to refold after unfolding [33,36,51]. However, their small size and lack of an Fc region can also result in rapid clearance and susceptibility to proteolytic degradation in complex biological environments [51]. In the context of tuberculosis, additional challenges arise from the extracellular and intracellular conditions encountered by *Mycobacterium tuberculosis*. These include exposure to proteases, oxidative stress, and variable pH within macrophages and granulomatous lesions. Such factors may affect nanobody persistence, functional half-life, and effective concentration at the site of infection. Moreover, the stability of nanobody-based constructs may be influenced by fusion partners, including antimicrobial peptides or enzymatic domains. While nanobodies can serve as robust targeting modules, the overall behavior of fusion constructs must be evaluated experimentally, as payload-induced instability or aggregation may limit functional efficacy. These considerations highlight the importance of assessing nanobody stability and persistence under physiologically relevant conditions, particularly in models that reflect the complex microenvironments associated with tuberculosis infection.

## 7. Conclusions and Future Perspectives

The Ag85 complex occupies a unique position at the intersection of mycobacterial physiology [7,32], immunogenicity [31], and therapeutic vulnerability [44,45]. As essential mycolyltransferases that are both secreted and cell wall-associated, Ag85A, Ag85B, and Ag85C represent rare examples of bacterial enzymes that are simultaneously indispensable for survival and accessible to extracellular binding molecules. While decades of research have established Ag85 as a dominant immune antigen and a validated small-molecule drug target, the potential of antibody-based strategies to modulate Ag85 function has remained largely unexplored. The analysis presented in this review suggests that this gap reflects not an inherent infeasibility, but rather the absence of selection paradigms explicitly designed to identify functionally relevant antibody binders.

Nanobodies offer a promising means to revisit Ag85 targeting from a functional perspective [35,37,50]. Their ability to engage conformationally restricted epitopes may enable modulation of enzymatic processes central to mycobacterial cell wall biosynthesis [4,6]. Within this framework, combinatorial strategies that incorporate nanobody-mediated sensitization may enhance the efficacy of existing antibiotics [20,44,45]. Their small size, structural rigidity, and ability to engage recessed or conformationally sensitive epitopes distinguish them from conventional antibodies and make them particularly well suited for probing enzymatic vulnerabilities. Importantly, nanobody-mediated effects on Ag85 need not result in complete enzymatic inhibition to be biologically meaningful. Partial interference with mycolyl transfer, enzyme localization, or cell wall dynamics may suffice to sensitize mycobacteria to antibiotics, environmental stress, or cell wall-degrading enzymes. Looking forward, the most realistic and impactful applications of Ag85-targeting nanobodies are likely to emerge within combinatorial frameworks. Rather than replacing existing antimicrobials, nanobodies may function as adjuvant agents that weaken the mycobacterial cell envelope and enhance the efficacy of antibiotics or phage-derived enzymes. Such strategies align with broader shifts in antimicrobial development toward sensitization, potentiation, and reduction in resistance pressure. In the context of tuberculosis, the intracellular lifestyle of *Mycobacterium tuberculosis* and its residence within macrophages introduce additional considerations for antibody-based approaches. While nanobodies are unlikely to achieve spontaneous cytosolic delivery, their favorable uptake by macrophages and potential localization within endosomal or phagosomal compartments may be biologically relevant and warrant systematic investigation. In parallel, pulmonary delivery represents a plausible route to enhance local exposure in the lung, although multiple biological and physicochemical barriers remain to be addressed. Future studies should prioritize integrative approaches that combine structural insight, functional screening, and physiologically relevant model systems. By aligning antibody discovery efforts with clear functional endpoints, it may be possible to transform Ag85 from a well-characterized immune antigen into a versatile node for antibody-mediated intervention against *Mycobacterium tuberculosis*.

Overall, this perspective reframes Ag85 not only as an immunodominant antigen but as a tractable node for functional intervention in mycobacterial cell wall biology.

## Figures and Tables

**Table 1:** Biological and immunological features of the Ag85 complex and implications for antibody-based targeting.

Ag85 Isoform	Primary Enzymatic Function	Localization	Immunogenicity	Evidence from Small-Molecule Inhibition	Accessibility to Antibodies	Implications for Nanobody Targeting	Key References
Ag85A (FbpA)	Mycolyltransferase; transfer of mycolic acids from TMM to trehalose and arabinogalactan	Cell wall-associated; secreted	High	Chemical and genetic inhibition disrupts cell wall assembly	Partial, context-dependent	Potential for allosteric interference or disruption of enzyme–envelope interactions	[7,8,9]
Ag85B (FbpB)	Major mycolyltransferase; dominant contributor to TDM synthesis	Abundantly secreted; cell envelope-associated	Very high (immunodominant)	Validated target of multiple small-molecule inhibitors	Relatively high	Prime candidate for functional nanobody modulation and sensitization strategies	[11,12,14]
Ag85C (FbpC)	Mycolyltransferase; contributes to maintenance of cell wall integrity	Predominantly cell-associated	Moderate to high	Inhibition affects envelope permeability and growth	More restricted	Targeting may enhance cross-family or synergistic effects	[4,8]

**Table 2:** Representative small-molecule inhibitors of the Ag85 complex: targets, mechanisms of interaction, and implications for antibody-based strategies.

Inhibitor/Compound Class	Target (Ag85 Isoform)	Mechanism of Interaction	Functional Effect on Ag85	Structural Insight	Implications for Nanobody Strategy	References
Ebselen	Primarily Ag85C (also Ag85A/B)	Covalent interaction with catalytic residues; stabilization of inactive conformation	Inhibits mycolyltransferase activity; disrupts cell wall biosynthesis	Crystal structure shows binding outside catalytic center and conformational trapping	Supports feasibility of non-active-site inhibition and allosteric modulation by nanobodies	[11]
Tetrahydrolipstatin (THL, Orlistat)	Ag85A and Ag85C	Covalent binding to catalytic serine in active site	Blocks mycolyl transfer reactions; reduces mycolate incorporation	Structural data confirm active-site targeting and covalent inhibition	Provides reference for direct catalytic inhibition; contrasts with potential steric/allosteric nanobody effects	[8]
Trehalose analogs/substrate mimetics	Ag85A/B/C	Competitive inhibition via mimicry of TMM or trehalose substrates	Interferes with TDM formation and substrate processing	Indicates accessibility of substrate-binding groove	Suggests targeting of substrate-binding regions by nanobodies	[9]
Broad-spectrum cell wall inhibitors affecting mycolate pathways	Indirect effect on Ag85 (via substrate availability)	Disruption of mycolic acid biosynthesis upstream of Ag85	Alters substrate pool and cell wall composition	Functional coupling between biosynthesis pathways	Highlights importance of indirect modulation and substrate dynamics	[10,20]
Structure-guided synthetic inhibitors	Ag85 family (A/B/C)	Binding to surface regions or allosteric sites	Partial inhibition; altered enzyme dynamics	Structural studies reveal non-catalytic binding sites	Reinforces concept of allosteric vulnerability exploitable by nanobodies	[11,19]

## Data Availability

Not applicable.
